# Weaning and stunting affect nitrogen and carbon stable isotope natural abundances in the hair of young children

**DOI:** 10.1038/s41598-020-59402-8

**Published:** 2020-02-13

**Authors:** Trenton Dailey-Chwalibóg, Jean-François Huneau, Véronique Mathé, Patrick Kolsteren, François Mariotti, Md. Rayhan Mostak, Md. Abdul Alim, Murad Md. Shamsher Tabris Khan, Md. Abdul Hashem Khan, Benjamin Guesdon, Helene Fouillet

**Affiliations:** 1 Université Paris-Saclay, AgroParisTech, INRAE, UMR PNCA, 75005 Paris, France; 2 Department of Food Technology, Safety and Health, Ghent University, Ghent, Belgium; 3 Department of Expertise and Advocacy, Action Contre la Faim (ACF), Paris, France; 4 Palli Karma-Sahayak Foundation (PKSF), Dhaka, Bangladesh; 5 Action Against Hunger (AAH), Dhaka, Bangladesh; 6 National Nutrition Service (NNS), Institute of Public Health Nutrition (IPHN), Dhaka, Bangladesh; 7 Community Based Health Care (CBHC), Dhaka, Bangladesh

**Keywords:** Epidemiology, Malnutrition

## Abstract

Natural abundances of stable nitrogen and carbon isotopes (δ^15^N and δ^13^C) can vary with both dietary intake and metabolic (specifically catabolic) state. In low-income countries, weaning is a period of dietary transition from milk to plant-based foods and a high-risk period for malnutrition. We explored how diet and malnutrition impact hair δ^15^N and δ^13^C in young children by an observational, cross-sectional study in Cox’s Bazar District, Bangladesh [255 children, 6–59 months with 19.6% wasted (7.1% severely) and 36% stunted (9.8% severely)]. Hair δ^15^N and δ^13^C exhibited exponential decreases with age, with the loss of one trophic level (3.3‰ and 0.8‰, respectively) from 6 to 48 months, which we associate with the shift from exclusive breastfeeding to complete weaning. After adjustment for age and breastfeeding status, hair isotopic values were unaffected by wasting but lower in severe stunting (−0.45‰ to −0.6‰, *P* < 0.01). In this population of young children, whose isotopic values in hair primarily depended on age, we failed to observe any effect of wasting, likely due to opposite, compensating effects between dietary and metabolic changes involved. In contrast, we evidenced low δ^15^N and δ^13^C values in severely stunted children that likely indicate chronic exposure to diets low in animal products.

## Introduction

The natural abundances of stable carbon and nitrogen heavy isotopes (δ^13^C and δ^15^N) in body proteins reflect those of the diet, plus a small discrimination factor, known as the trophic step, which is due to the preferential elimination of light isotopes and is smaller for carbon (+1‰) than for nitrogen (+3–4‰)^1,2^. Since δ^13^C and δ^15^N values in the body primarily reflect those of the diet, they have long been used in ecology and archaeology to reconstruct trophic webs and characterize ancient diets^3–6^. More recently, they have been used in epidemiology as biomarkers to assess the consumption of fish, animal products, and sugar^7–13^. In this regard, measuring isotopic natural abundances in hair has several advantages. Hair is easily and non-invasively sampled and its isotopic values correlate well with those in other body proteins. Moreover, hair keratin is an ideal isotopic archive because it is produced sequentially and is inert after synthesis^14,15^, provided that a sufficient number of follicles is sampled to limit the potential bias due to the presence of a small proportion of hair in the telogen phase, i.e. not growing^15–17^. Considering an average hair growth rate of 1 cm per month^17,18^ and a one-week delay between hair synthesis and bulb exit^19^, the 5 mm of hair closest to the scalp contains isotopic information for the period between −3 and −1 weeks before sampling.

Moreover, it is now recognized that the isotopic discrimination between the body and the diet (i.e., the trophic step) varies between subjects according to their particular metabolic orientation, leading to changes in δ^13^C and δ^15^N in body proteins. For example, an anabolic shift, as observed during pregnancy, is associated with a decrease in hair δ^15^N due to a decrease in the nitrogen trophic step^20^. Conversely, a catabolic shift with weight loss, as observed during anorexia nervosa or in women experiencing nausea during early pregnancy, has been associated with an increase in δ^15^N and a decrease in δ^13^C in hair^14,15,21^. In rats submitted to prolonged caloric restriction with maintained protein intake, we recently evidenced tissue δ^15^N and δ^13^C variations resulting from metabolic adaptations; in particular, we observed that increased amino acid orientation towards the transamination and deamination pathways fueling gluconeogenesis, and the mobilization of muscle ^15^N-enriched amino acids, resulted in an increase in δ^15^N^22^. Thus, beyond simple markers of dietary exposure, hair δ^13^C and δ^15^N measurements could also be used to identify a catabolic state characterized by the mobilization of body proteins for new protein synthesis, which is believed to be part of the natural history of acute malnutrition in children.

Early childhood is a period of dietary transition, with the gradual shift from infant-specific, milk-based feeding to family foods. In low-income countries, weaning is known to be a high-risk period for malnutrition, in particular because of the introduction of inadequate complementary foods with low protein content and low energy density. In these countries, poor sanitary conditions are associated with a high risk of infection during early childhood, which further increases the risk of malnutrition^23–27^. Malnutrition can occur in two forms: (1) wasting (i.e., acute malnutrition) is a rapid loss of fat and muscle mass due to recent food deprivation and/or disease; (2) stunting (i.e., chronic malnutrition) is the consequence of a progressive alteration in linear growth, caused by long-term insufficient nutrient intake and recurrent infections. Stunting has been recently described as a consequence of repeated episodes of wasting^28^. Although archaeologists frequently use δ^13^C and δ^15^N measurements in hair or bone collagen to identify weaning practices in ancient civilizations^29–32^, few data are available on the evolution of δ^13^C and δ^15^N during this period in contemporary populations^33,34^. Additionally, to what extent hair δ^13^C and δ^15^N values are affected by wasting or stunting during the weaning period remains to be defined.

In this context, the aim of the present study was to assess the association between hair δ^13^C and δ^15^N, the diet and the severity of wasting or stunting in a representative population of young (6–59 months) children in Bangladesh.

## Results

### Population description

The final number of children with hair isotopic measurements was 255, with a mean age of 33.7 months, a sex ratio close to 50% and approximately one third of the population partially or exclusively breastfed (Table 1). Moreover, ~20% of the population was wasted (~7% severely) and ~36% was stunted (~10% severely). The characteristics of our study population were similar to those of the representative sample of children surveyed during the overarching nutritional survey (n = 654), from which our population was randomly sub-sampled (Supplementary Table S1), except for a higher proportion of children with severe acute malnutrition (7.1% vs. 4.3%).

**Table 1:** Main characteristics of the population.

	Population characteristics (n = 255)
Age (months)	33.7 ± 15.4
Height/length (cm)	86.7 ± 10.8
Weight (kg)	11.2 ± 2.6
Female	120 (47.1%)
Breastfed	85 (33.3%)
Wasted	50 (19.6%)
Severely wasted	18 (7.1%)
Stunted	93 (36.5%)
Severely stunted	25 (9.8%)

Values are means ± SD or n (%).

In accordance with the respective anthropometric criteria by which they were diagnosed, wasted children had a lower mid-upper arm circumference (MUAC) and/or weight-for-height Z-score (WHZ) than non-wasted children, while stunted children had a lower height-for-age Z-score (HAZ) than non-stunted children (Table 2). In addition, compared to non-wasted children, wasted children were also younger, more often boys and fully or partially breastfed, and had a lower dietary diversity score (DDS), a lower HAZ and a lower weight-for-age Z-score (WAZ). In contrast, compared to non-stunted children, stunted children were similar in terms of age, sex ratio and DDS but had a lower WAZ and MUAC (for severely stunted children only).

**Table 2:** Characteristics of children with wasting or stunting.

	No wasting (n = 205)	Wasting (n = 50)	Severe wasting (n = 18)
Female	103 (49.7%)	17 (34.0%)*	8 (44.4%)
Weaned	145 (70.7%)	25 (50.0%)*	7 (38.9%)*
Age (months)	34.6 ± 14.9	29.4 ± 16.7	25.5 ± 0.17.0*
DDS	4.7 ± 1.6	4.1 ± 1.7	3.7 ± 2.0*
WAZ	−1.20 ± 0.96	−2.88 ± 0.87*	−3.10 ± 1.07*
HAZ	−1.48 ± 1.13	−1.93 ± 1.50*	−1.43 ± 1.67
MUAC (mm)	147.0 ± 10.3	125.9 ± 11.1**	120.8 ± 15.0**
WHZ	−0.51 ± 0.90	−2.52 ± 0.68**	−3.13 ± 0.57**
Stunting	70 (34%)	23 (46.0%)	5 (27.8%)
	**No stunting (n = 162)**	**Stunting (n = 93)**	**Severe stunting (n = 25)**
Female	76 (46.9%)	44 (47.3%)	13 (52.0%)
Weaned	104 (64.2%)	66 (70.9%)	18 (72.0%)
Age (months)	32.2 ± 16.6	36.1 ± 12.8	35.9 ± 13.1
DDS	4.5 ± 1.8	4.6 ± 1.5	4.8 ± 1.4
WAZ	−1.14 ± 1.12	−2.21 ± 0.87**	−2.80 ± 0.90**
HAZ	−0.92 ± 0.91	−2.73 ± 0.74**	−3.71 ± 0.83**
MUAC (mm)	144.3 ± 14.2	140.5 ± 11.7	137.0 ± 15.7*
WHZ	−0.84 ± 1.21	−0.96 ± 1.09	−0.96 ± 1.15
Wasting	27 (16.6%)	23 (24.7%)	9 (36.0%)

DDS, dietary diversity score; WAZ, weight-for-age Z-score; HAZ, height-for-age Z-score; MUAC, mid-upper arm circumference; WHZ, weight-for-height Z-score. Values are means ± SD or n (%). **and *, significant difference versus control (*P* < 0.01 and *P* < 0.05, respectively). Children with severe wasting or severe stunting represent sub-populations of children with wasting or stunting, respectively. The same 255 children were categorized either according to their level of wasting or their level of stunting. The results of these two categorizations correspond to the two horizontal blocks of the table.

In bivariate logistic regression, age was weakly and negatively associated with the risk of wasting while still being breastfed was a strong determinant for wasting. Neither age nor weaning status were associated with the risk of stunting (Supplementary Table S2).

### Hair δ^15^N and δ^13^C: links with anthropometric parameters

The measured C/N ratios in hair (3.14 ± 0.17, mean ± SD) were close to the theoretical value of keratin^35^ (confirming that sample preparation, i.e., cleaning and delipidation, was carried out with care) and were not statistically different across conditions (breastfeeding, wasting or stunting).

For the 255 children included, hair δ^15^N and δ^13^C values were 8.74 ± 1.25‰ and −22.18 ± 0.75‰ (mean ± SD), respectively, and did not vary between boys and girls (Supplementary Table S3). For the 7 exclusively breastfed children (breastfed and DDS = 0), the δ^15^N and δ^13^C values (11.45 ± 0.69‰ and −22.01 ± 0.44‰, respectively) were significantly higher compared to those of the 170 weaned children (8.19 ± 0.78‰ and −22.32 ± 0.76‰, Supplementary Table S3). Both δ^15^N and δ^13^C were negatively correlated with age, height and weight (Table 3). Hair δ^15^N was also negatively correlated with head circumference and MUAC. Moreover, both δ^15^N and δ^13^C were positively correlated with HAZ.

**Table 3:** Pearson correlation coefficients between natural abundances of stable nitrogen and carbon isotopes in hair (δ^15^N and δ^13^C), and age and anthropometric indicators (n = 255).

	δ^15^N	δ^13^C
Age	−0.63***	−0.32***
Height	−0.60***	−0.23**
Weight	−0.55***	−0.24***
MUAC	−0.29***	−0.08
Head circumference	−0.34***	−0.12
BMI	0.11	0.01
HAZ	0.21**	0.22**
WHZ	−0.06	−0.07

MUAC, mid-upper arm circumference; HAZ, height-for-age Z-score; WHZ, weight-for-height Z-score. ***P < 0.0001, **P < 0.01.

### Hair δ^15^N and δ^13^C: trajectories during weaning

**Figure 1:**
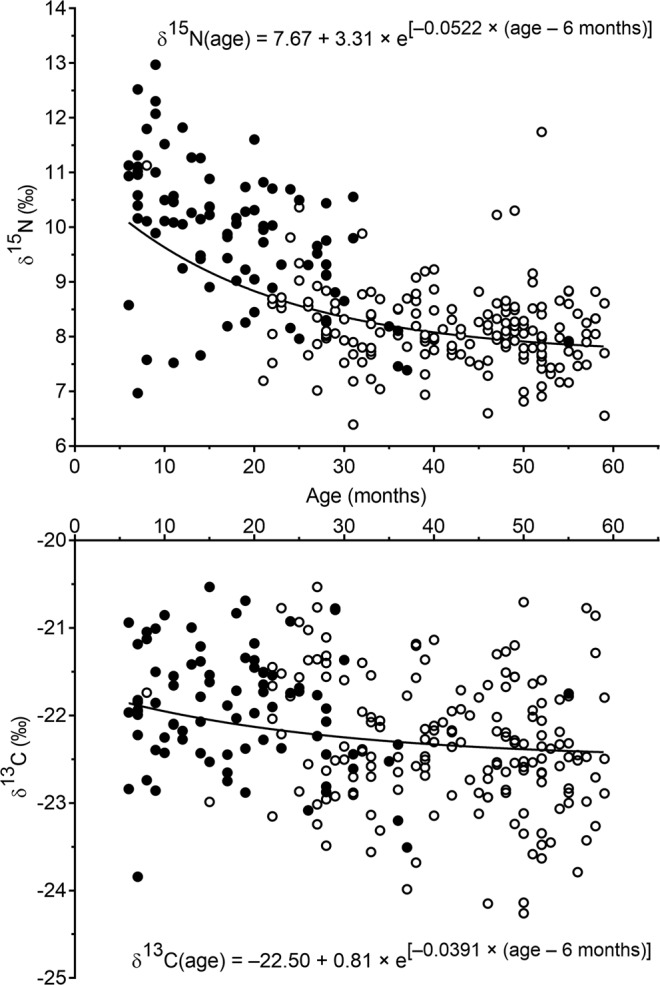
δ^15^N and δ^13^C in hair as a function of age in children aged 6 to 60 months (n = 255). Empty circles represent children who were fully weaned and filled circles represent children who were partially or exclusively breastfed.

Plotting δ^15^N or δ^13^C against age showed an exponential decay between 6 and 60 months (Fig. 1). These decreases were fit using the following 3-parameter equation:

$$\delta X({\rm{a}}{\rm{g}}{\rm{e}})={\delta X}_{{\rm{\infty }}}+\Delta \times {{\rm{e}}}^{-{\rm{k}}\times ({\rm{a}}{\rm{g}}{\rm{e}}-6{\rm{m}}{\rm{o}}{\rm{n}}{\rm{t}}{\rm{h}}{\rm{s}})}$$

where *X* stands for either ^15^N or ^13^C, δ*X*_∞_ (‰) represents the plateau δ^15^N or δ^13^C value at the final isotopic steady state (i.e. the expected value in adults), Δ (‰) is the difference between the δ^15^N or δ^13^C value measured at 6 months of age and the corresponding expected value in adults (i.e., the weaning trophic level decrease), and k (%/month) represents the rate of decrease in δ^15^N or δ^13^C (i.e., the isotopic turnover rate).

Because the dispersion of δ^13^C values at a given age was large compared to the variation of δ^13^C with age, a two-step procedure was used to estimate the 3 parameters for ^13^C. The δ^13^C_∞_ value was first estimated as the mean δ^13^C value for fully weaned children ≥48 months of age (our closest proxy for the value in adults) and this value was then forced into the model to estimate the other two parameters.

The weaning trophic level decrease (Δ) was much higher for δ^15^N than for δ^13^C (3.31‰ vs. 0.81‰) but the isotopic turnover rate (k) rate was similar for the two isotopes (Table 4). The time needed to reach 90% of the final isotopic equilibrium $(t0.9=-\,{\rm{l}}{\rm{n}}(1-0.9)/\text{k})$ was 52 months, from the initial age of 6 months, on average, for both isotopes.

**Table 4:** Parameter estimates [95% confidence intervals] for δ^15^N and δ^13^C fitted trajectories during weaning (exponential decay between 6 and 60 months of age).

	δ^15^N		δ^13^C	
δ*X*_∞_ (‰)	7.67	[7.21–8.13]	−22.50	[−22.70–22.30]
Δ (‰)	3.31	[2.85–3.76]	0.81	[0.56–1.05]
k (%/month)	5.22	[3.05–7.39]	3.91	[2.20–5.62]

δ*X*_∞_, final isotopic steady state; Δ, weaning trophic level decrease; k, isotopic turnover rate.

After adjustment for age, δ^15^N was lower in fully weaned children compared to children fully or partially breastfed (8.42 ± 0.08 vs. 9.39 ± 0.13, *P* < 0.0001) while no difference was observed for δ^13^C, indicating that at least for δ^15^N, the effects of age and breastfeeding status were not statistically confounded.

### Hair δ^15^N and δ^13^C: effects of wasting and stunting

**Figure 2:**
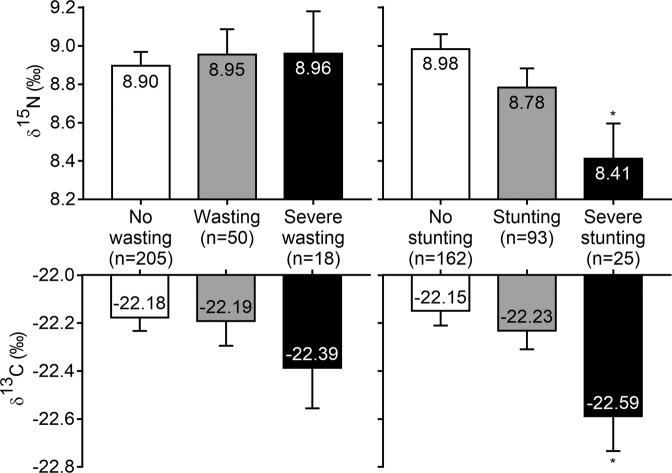
Effect of wasting and stunting on age- and breastfeeding-adjusted δ^15^N and δ^13^C in hair of children (6–60 months). Data are least-square means ± SE. *Different from control (non-wasted or non-stunted) children, *P* < 0.01.

Before adjustment for age and breastfeeding status, hair δ^15^N was higher in wasted children and lower in stunted children compared to non-wasted and non-stunted children, respectively. Hair δ^13^C was also lower in severely stunted children compared to non-stunted children (Supplementary Table S3). After adjustment for age and breastfeeding status, there was no difference in hair δ^15^N and δ^13^C between wasted children and their controls. However, both δ^15^N (−0.57‰, *P* < 0.01) and δ^13^C (−0.44‰, *P* < 0.01) were significantly lower in stunted children (Fig. 2).

In bivariate logistic regression, δ^15^N but not δ^13^C was weakly and positively associated with the risk of being wasted. This association was no longer observed in multi-adjusted models. In contrast, δ^15^N, and to a lesser extent δ^13^C, were negatively associated with the risk of being stunted. In multi-adjusted models, δ^15^N was still negatively associated with the risk of being severely stunted whereas the association was marginally significant for δ^13^C (Supplementary Table S4).

## Discussion

In the present study, we showed, with high statistical power due to the large sample of children, that hair δ^15^N and δ^13^C values decreased between 6 months and 4 years of age, with little variation thereafter—a consequence of the weaning process and the gradual switch from breast milk to plant-based family meals of lower δ^15^N and δ^13^C values^11,36^. In this population of young children, we failed to observe any association between hair δ^15^N and δ^13^C and wasting, but highlighted that both were associated with stunting, a new result that has not been reported so far. Indeed, compared to non-stunted children, severely stunted children exhibited lower hair δ^15^N (−0.6‰) and δ^13^C (−0.4‰) values that likely reflect chronic exposition to diets low in animal source food (ASF).

One of the most important results of this study is our report of an exponential decrease in hair δ^15^N and δ^13^C between 6 months and 5 years of age, which was more pronounced for ^15^N than for ^13^C. Although many archaeological studies rely on the hypothesis of a decrease in protein δ^15^N and δ^13^C during weaning to reconstruct the practices of ancient civilizations^6,29–31,37,38^, there are very few data on the evolution of hair δ^15^N and δ^13^C with age in contemporary children. Fogel *et al*., using periodic fingernail clippings sampled from mother-infant pairs, were among the first to report a δ^15^N increase between birth and 6 months of age due to breastfeeding, followed by a gradual δ^15^N decrease after weaning initiation^33^. More recently, and again using fingernail clippings from mother-infant pairs collected during breastfeeding *versus* formula-feeding, Fuller *et al*. reported that breastfeeding resulted in gradual increases in both δ^15^N and δ^13^C until weaning onset, when infants’ enrichments (relative to their mothers’) reached 2–3‰ for ^15^N and a 1‰ for ^13^C, followed by gradual decreases during weaning, which were more pronounced for ^15^N than for ^13^C^34^. However, these two studies remain largely qualitative, as the limited number of participants did not allow for statistical analysis, and the follow-up periods were restricted to the early phase of weaning (up to 1.5 years of age) and not to complete weaning. Even though we did not measure hair δ^15^N and δ^13^C values in mothers, nor in children under 6 months of age, our data shed light on the impact of breastfeeding and weaning on δ^15^N and δ^13^C variations during infancy and early childhood (up to 5 years of age). Firstly, aside from age, we observed that hair δ^15^N and δ^13^C were higher in fully breastfed children than in weaned children no longer receiving breast milk. Secondly, analysis of hair δ^15^N and δ^13^C kinetics between 6 months and 5 years of age supports the hypothesis of an underlying trophic level effect. Considering that the isotopic values at the plateau correspond with those of adults, and assuming that data from children at 6 months of age are a proxy for the maximum values reached at the end of breastfeeding period, the Δ parameters in our equations correspond to the maximum isotopic enrichment in infants relative to their mothers (i.e., the breastfeeding trophic level shift equivalent to the weaning trophic level decrease). The second assumption is supported by the lack of difference between our model predictions (by curve-fitting) for δ^15^N and δ^13^C at 6 months and our observations in the 7 exclusively breastfed children in our study population. Our estimates for Δ (i.e., 3.3‰ [2.8–3.8] for ^15^N and 0.8‰ [0.6–1.0] for ^13^C) correspond with the magnitude of a trophic level shift reported in the literature (i.e., 3–4‰ for ^15^N and 1‰ for ^13^C)^36^. Therefore, our data support the hypothesis that after birth, exclusive breastfeeding induces gradual increases in δ^15^N and δ^13^C in infants compared to their mothers, up to a difference on the order of one trophic level around the age of 6 months. These increases are then reversed during weaning, as the proportion of breastmilk in the infant diet is gradually reduced. A state close to isotopic equilibrium with the family diet is reached around 4 years of age, with little change in δ^15^N and δ^13^C afterwards. ^13^C and ^15^N bioaccumulation, which leads to Δ^13^C and Δ^15^N trophic steps, is in large part due to discrimination against these heavy isotopes during the formation of respiratory CO_2_ and N waste. Δ^15^N has been shown to vary slightly based on the quality of dietary protein and efficiency of its anabolic use^39,40^. This effect of dietary protein quality is sometimes confused with the effect of the δ^15^N value of the diet, as plant proteins, compared to animal proteins, are of poorer nutritional quality and lower δ^15^N values. The nutritional quality of breast milk protein is characteristic of the species and is independent of maternal diet quality^41^. Because of this, the Δ^15^N trophic step between children’s hair and their mother’s breast milk is independent of the quality of the family diet and its δ^15^N value. Therefore, the magnitude of decrease in δ^15^N and δ^13^C we observed in children during weaning is applicable to all current and past dietary conditions, regardless of their nature and quality, provided that children are weaned on the same diet as their mothers.

While the dietary transition associated with weaning is undoubtedly the underlying cause of the δ^15^N and δ^13^C decrease with age, the effects of age and breastfeeding status were not fully confounded in our study, at least for δ^15^N, for two reasons. First, we assume that in our study, breastmilk consumption varied in partially breastfed children—and children were likely to receive less and less breast milk with age. Second, after a diet transition, hair progressively integrates the isotopic signature of the new diet because endogenous amino acids released during the renewal of body proteins (mainly muscle) represent a significant supply to the keratin precursor pool^42^; at the time of dietary transition, these endogenous amino acids still carry the isotopic signature of the former diet. The isotopic differences between endogenous and exogenous amino acids mitigate the signature of the new diet detectable in hair, at least until a new isotopic steady state is reached in muscle tissue^42^. It should be also noted that, in our study, the ratio between the SD of the isotopic abundance for the whole population and the predicted weaning trophic level was higher for ^13^C (0.93) compared to ^15^N (0.38). This indicates that our exponential models left more variability unexplained for δ^13^C than for δ^15^N. The significant variability in δ^13^C (in children of all ages) likely reflects variability in the proportion of ^13^C-rich C4 plants in the family diets in our sample. Rice (a C3 plant) is the staple food consumed by resource-poor Bangladeshis. Yet, some families may have received food aid in the form of corn (a C4 plant). However, we do not know the proportion of corn consumption in the diet of each family. In fully or partially breastfed children, the proportion of C4 plants in the family diet has an indirect effect by modifying the level of ^13^C enrichment of breast milk. Last, it is also worth noting that in our study, δ^15^N and δ^13^C estimates at the plateau were lower than those previously reported by Fuller *et al*. in adults^34^, but close to those previously reported for modern vegetarians and vegans^5,12^—suggesting a low consumption of ASF in this low-income Bangladeshi population^43^. Regarding hair δ^15^N, the adult values estimated by our δ^15^N trajectory fit are very close to those reported for a rural Kenyan population whose livelihood also relies on agriculture^44^. However, hair δ^13^C values notably differ between these two populations, due to differential consumption of foods derived from C4 plants. Corn and millet (C4 plants) are the major cereals in the Kenyan diet^44^ whereas the major staple food in Bangladesh is rice (C3)^45^.

Assuming that isotopic variations in δ^15^N and δ^13^C values between 6 months and 5 years reflect a progressive dietary shift during the weaning process, the correlations between these isotopic values and age-related parameters (e.g., height, weight, head circumference, and MUAC) are likely indirect consequences of this diet shift as well. This explanation is consistent with the fact that hair δ^15^N and δ^13^C values were not correlated with anthropometric parameters that are weakly or not related to age (e.g., BMI, WHZ or WAZ) with the notable exception of HAZ. HAZ was found to be weakly and positively correlated with both δ^15^N and δ^13^C. Indeed, δ^15^N and δ^13^C values were significantly lower in severely stunted children (HAZ < −3) compared to control children (HAZ ≥ −2), even after adjusting for age and breastfeeding status. Despite strong statistical significance (*P* < 0.01), these effects remain small compared to the trophic level decrease during weaning, especially for δ^15^N. Consequently, the risk of archaeologically mischaracterizing a severely stunted child for a child in process of weaning is limited, but non-negligible. Conversely, in a contemporary population where children’s ages and breastfeeding statuses are known, δ^15^N and δ^13^C measurements in hair could help identify those with severe chronic malnutrition. Such low isotopic values observed in severely stunted children could be due to the consumption of a diet of low isotopic value by either: (1) children after weaning; and/or (2) their mothers during pregnancy and lactation. A dietary explanation is likely, since diets consisting mainly of plant protein (i.e., poor in ASF) have low δ^15^N and δ^13^C values^46^, and very low dietary diversity with almost no ASF consumption has been identified as a risk factor for stunting^47^. In the present study, we found no difference in dietary diversity scores (DDS) between severely stunted and non-stunted children. However, DDS assessment was based on caretaker declaration and did not take into account the quantities consumed for each of the groups declared. Therefore, it is not possible to rule out a lower ASF consumption in severely stunted children compared to non-stunted children. In this regard, hair δ^15^N and δ^13^C measurements could be used as alternatives to assess sub-optimal breastfeeding practices, DDS and ASF consumption which occurred in the past, with a lower risk of bias. Alternatively, the early origin of severe stunting could also constitute a possible explanation for the low values of δ^15^N and δ^13^C. Intrauterine growth retardation (IUGR) linked to the poor nutritional status of mothers is recognized as a risk factor for severe stunting^25,48^. Low dietary diversity in pregnant women could lead to IUGR and low δ^15^N and δ^13^C values in newborns, although experimental evidence of this is not available to date. These low values at birth may, in part, explain the low values observed after 6 months of age in our population. These two potential causes are not mutually exclusive and could both contribute to the low hair δ^15^N and δ^13^C values measured in severely stunted children.

Quite surprisingly, in this study, we failed to observe a specific isotopic signature for wasted children. Yet, it has been previously reported that muscle wasting in anorexia was associated with an increase in hair δ^15^N^14,15^ because of a higher trophic step due to a metabolism more oriented towards catabolism. A first possible explanation is that, in our population, because acute malnutrition mostly occurred between 6 and 24 months of age, at a period of rapid decline in δ^15^N in hair due to the introduction of plant foods during weaning, any δ^15^N increase due to muscle wasting was blurred by an antagonistic δ^15^N decrease due to weaning. Wasted children, just like stunted children, are likely to be weaned on a diet of poor nutritional quality, almost devoid of ASF, and become wasted as a consequence of recent diseases or deprivation. The hypothesis of an antagonistic effect of diet quality and muscle wasting on natural isotopic abundances in hair is supported by the observation that, compared to children with severe stunting, children with severe wasting have similar low δ^13^C values that are characteristic of ASF-poor diets, but higher δ^15^N values. In addition, it has been shown that in children with non-edematous acute malnutrition, food deprivation is not associated with an increase in body protein breakdown beyond that measured in well-nourished children^49^. The mechanisms responsible for wasting are thus quite different from those involved in weight loss in adolescents or adults with anorexia, whose lean body mass is initially much higher than that of children. These two hypotheses could explain why, unlike in anorexia in adolescents and adults, acute malnutrition in children is not associated with an increase in hair δ^15^N.

The main strength of this study is the sample size and age distribution that, for the first time, models the evolution of δ^15^N and δ^13^C across the entire weaning period in humans. In addition, the high prevalence of chronic malnutrition in our population provides sufficient power to highlight its isotopic signature in hair. This study has also some limitations. Information on children’s food consumption is limited, qualitative and caretaker-reported. Additionally, we did not measure the natural ^15^N and ^13^C abundances in locally consumed staple foods. This limits our ability to ascertain the links between hair δ^15^N and δ^13^C values and dietary diversity and ASF consumption. Lastly, since each 5 mm segment corresponds to a 2-week period, it would have been interesting to have isotopic measurements over a greater number of consecutive segments along the hair follicle—to trace each child’s nutritional history and to highlight, at the individual level, the isotopic trajectory related to dietary transition during weaning, and its possible deviation by an episode of acute malnutrition.

## Methods

### Ethical considerations

Ethical approval was obtained from the Ethics Committee at the University Hospital of Antwerp and the University of Antwerp (B300201627243) and the National Research Ethics Committee (NREC) at the Bangladesh Medical Research Council (BMRC) (BMRC/NREC/2016–2019/1463). All parents and/or legal guardians of participants were asked to sign an informed consent form and those who were illiterate indicated consent by inked thumbprint in the signature space. All research described in this manuscript was performed in accordance with relevant guidelines and regulation.

### Population

This observational, cross-sectional study was conducted on a sub-sample of children randomly drawn from a larger nutritional survey following the Standardized Monitoring and Assessment of Relief and Transitions (SMART) methodology and conducted in Ukhiya and Teknaf Upazillas, in the Cox’s Bazar District, Bangladesh. Sample size was calculated based on the SMART two-stage sampling methodology, using ENA software, in order to produce a precise estimate of the prevalence of Global Acute Malnutrition (GAM, defined as WHZ < −2 SD and/or bilateral pitting edema)^50^. A total of 1,095 households and 728 children were reached in the SMART survey; in this study, only 665 caretakers agreed that their children participate in the stable isotope analysis (SIA) sub-study, of which only 655 children had hair at the time of the investigation. A random sample of 255 children was selected from these 665 participants for isotopic analysis. However, because the number of severely wasted children in the SMART study was very low (28 or 4.3%, Supplementary Table 1), we decided to oversample this category of children to reach a sufficient group size to allow for statistical comparisons. Therefore, all severely wasted children recruited in the SMART survey with enough hair to perform isotopic analyses (n = 18) were included in the final sample. The prevalence of stunting and wasting in the final sample were close to those measured in the SMART survey, conducted on a representative sample of children in Cox’s Bazar, and close to the prevalence of the joint malnutrition estimates for south Asia issued by UNICEF/WHO/World Bank in 2019^51^.

### Data handling

All data were collected using structured questionnaires written in Bengali, and information was recorded on childhood morbidity and diet from immediate caregivers, which were usually mothers. The dietary diversity score (DDS) was calculated based on 7 food groups (grains, roots and tubers, legumes and nuts, dairy products, flesh foods, eggs, vitamin A-rich fruits and vegetables, other fruits and vegetables) by summing the number of unique food groups eaten by the child during the last 24 hours. Breastfeeding status was assessed based on current breastfeeding and DDS over the past 24 hours, as follows: (1) exclusive breastfeeding: currently breastfed and DDS = 0; (2) partial breastfeeding: currently breastfed and DDS > 0; (3) fully weaned: not currently breastfed. Anthropometric measurements were conducted by trained investigators based on WHO standardized procedures^52^. Naked or minimally clothed children were weighed to the nearest 0.1 kg using portable Salter spring scales while recumbent length or standing height was measured to the nearest 0.1 cm using standard UNICEF height boards. MUAC was measured on the left arm to the nearest 1 mm using a standard MUAC tape. Data were recorded on a mix of hard-copy, paper print-outs (then entered into EPI Info and Microsoft Excel) and tablets using Open Data Kit (ODK). Anthropometric indices (i.e., BMI, HAZ, WAZ, and WHZ) were calculated using WHO Growth Standards^53^. Stunting (chronic malnutrition) was defined as HAZ < −2 SD and severe stunting as HAZ < −3 SD. Wasting (acute malnutrition) was defined as WHZ < −2 SD or MUAC < 125 mm and severe wasting as WHZ < −3 SD or MUAC < 115 mm.

### Hair collection and measurement of isotopic natural enrichments

In these children, a sample of 50 hair follicles was covered in hair gel, twisted into a lock and cut as close to the patient’s scalp as possible with sharp, dissecting scissors. The gelled lock was then taped to a sheet of paper and the cut end was labeled and stored at room temperature before further analysis.

Two consecutive segment of 5 mm on the cut end of the lock were removed and transferred into Eppendorf tubes. Hair gel was removed by two consecutive immersions in 750 µL of ultrapure water for 2 minutes with gentle agitation. Hair samples were then delipidated in 1.5 mL of ether/ethanol (1:1) for 30 minutes, washed in 1.5 mL of ultrapure water for 2 minutes, delipidated once more for 30 minutes, and finally washed thrice consecutively for 2 minutes each, all while being gently agitated. Samples were dried overnight before being transferred into 2 mg tin capsules and analyzed by elemental analysis/isotope-ratio mass spectrometry, using an elemental analyzer (EA Vario Micro Cube, Elementar, Germany) coupled with an isotope-ratio mass spectrometer (Isoprime, VG instruments, Manchester, UK). Tyrosine (δ^15^N = 10‰ and δ^13^C = −23.20‰) was used for calibration and drift correction. The natural abundances of ^15^N and ^13^C in hair were expressed relative to standards (atmospheric N_2_ for ^15^N/^14^N and Vienna Pee Dee Belemnite for ^13^C/^12^C) using the delta notation, according to the following equation: δ (‰) = 1000 × (R_sample_ − R_standard_)/R_standard_, where R_sample_ and R_standard_ stand for the ratio between heavy and light isotope (^15^N/^14^N and ^13^C/^12^C) in the sample and standard, respectively. The measurement precision was very good, as assessed by repeated measurements of standards with SD of 0.07‰ for both δ^15^N and δ^13^C.

### Data analysis

All statistical analyses were performed using statistical analysis software (SAS 9.4, Cary, NC, USA). Unless otherwise specified, all data are presented as means ± SD or as n (frequencies). The only exceptions are data adjusted for age and/or breastfeeding status. In this case, we present least-square means estimated from linear models where age and/or breastfeeding status were included as covariates, and their associated standard error (that estimate the accuracy of the estimation of these least-square means). Frequency comparisons between non-stunted/wasted children (controls) and (severely) stunted/wasted children were conducted using χ^2^ tests. Pearson bivariate correlations were used to assess the associations between isotopic measurements and the anthropometric characteristics of subjects. Bivariate and multivariate logistic regression was used to assess the link between age, weaning status, δ^15^N and δ^13^C and the odds of being (severely) stunted or wasted. The Gauss-Newton method was used for non-linear adjustment of δ^15^N and δ^13^C with age. The effect of (severe) stunting or wasting on continuous variables was assessed using analysis of variance with post-hoc Tukey-Kramer tests. Regarding the effect of (severe) stunting or wasting on δ^15^N and δ^13^C, age and breastfeeding status were included as covariates in the model as they are major determinants of hair δ^15^N and δ^13^C. The validity of covariance analyses was verified by inspecting the normality of model residuals using the Shapiro-Wilk tests. Significance tests were two-sided and performed at the α = 0.05 or α = 0.01 levels.

Statistical analyses for δ^15^N and δ^13^C were performed using isotopic data obtained from the 5 mm hair segment closest to the scalp and the mean of the two adjacent 5 mm segments closest to the scalp. These two analyses lead to very similar numerical results and identical conclusions. Therefore, only results from the analysis of isotopic data on the 5 mm segment closest to the scalp are presented in this article. A mixed model for repeated data was also used to analyze the evolution of δ^15^N and δ^13^C along the hair follicle and look for a possible influence of stunting or wasting on this evolution. This analysis and its main results are described in the Supplementary analyses section of Supplementary Material.

## Supplementary information

Supplementary material.

## Data Availability

Individual data on age, sex, height, weight, mid-upper arm circumference, breastfeeding status as well as δ^15^N, δ^13^C, and the C/N ratio of each hair segment (proximal & distal) are available in the Supplementary Table S7. The use of these data must only be made with reference to the present publication. Other data are available from the corresponding author on reasonable request.
