# Supplementary material for: Weaning and stunting affect nitrogen and carbon stable isotope natural abundances in the hair of young children
Source: Sci Rep. 2020 Feb 13;10:2522. doi: 10.1038/s41598-020-59402-8 (PMC7018826; doi:10.1038/s41598-020-59402-8)
Supplement: Supplementary file 1 — Supplementary material. [file 41598_2020_59402_MOESM1_ESM.pdf]

Weaning and stunting affect nitrogen and carbon stable isotope natural abundances in the hair of young children

Trenton Dailey-Chwalibóg†[1,2,3,] , Jean-François Huneau†*[1] , Véronique Mathé[1], Patrick Kolsteren[2], François Mariotti[1], Md. Rayhan Mostak[4,5], Md. Abdul Alim[6], Murad Md. Shamsher Tabris Khan[6], Md. Abdul Hashem Khan[7], Benjamin Guesdon[3], Helene Fouillet*[1]

1 Université Paris-Saclay, AgroParisTech, INRAE, UMR PNCA, Paris, France

2 Department of Food Technology, Safety and Health, Ghent University, Belgium

3 Department of Expertise and Advocacy, Action Contre la Faim (ACF) Paris, France

4 Palli Karma-Sahayak Foundation (PKSF), Dhaka, Bangladesh

5 Action Against Hunger (AAH), Dhaka, Bangladesh

6 National Nutrition Service (NNS), Institute of Public Health Nutrition (IPHN), Dhaka, Bangladesh

7 Community Based Health Care (CBHC), Dhaka, Bangladesh

†These authors contributed equally.

*Author to whom correspondence should be addressed (jean_francois.huneau@agroparistech.fr and/or helene.fouillet@agroparistech.fr).

Supplementary Table S1: SMART sample description.

Variable	Sample size	
**Age (months)**	654	30.7 ± 15.6
**Height-length (cm)**	655	84.5 ± 11.0
**Weight (kg)**	655	10.9 ± 2.6
**Female**	656	318 (48.5%)
**Breastfed**	653	265 (40.6%)
**Wasted**	656	104 (15.8%)
**Severely wasted**	656	28 (4.3%)
**Stunted**	656	243 (37.0%)
**Severely stunted**	656	76 (11.6%)

Values are means ± SD or n (%)

Supplementary Table S2: Effect of age and breastfeeding status on the risk of being wasted or stunted.

	Age (months)		Breastfeeding status	
	OR	*P*	OR	*P*
Wasted	0.98 [0.96-1.00]	< 0.05	2.42 [1.29-4.56]	< 0.01
Severely wasted	0.96 [0.93-0.99]	< 0.05	3.80 [1.42-10.76]	< 0.01
Stunted	1.02 [1.00-1.03]	NS	0.73 [0.42-1.27]	NS
Severely stunted	1.02 [0.99-1.04]	NS	0.70 [0.26-1.70]	NS

OR, odds ratio [95% CI]. Breastfeeding status was assessed based on caregiver declaration. For breastfeeding status, two categories were considered: children exclusively or partially breastfed (breastfeeding status = 1) and fully weaned children (breastfeeding status = 0).

Supplementary Table S3: Effects of sex, breastfeeding status, wasting and stunting on the raw (unadjusted) $\delta^{15}N$ and $\delta^{13}C$ data.

	$\delta^{15}N$	$\delta^{13}C$
	**Sex**	
**Boys (n=135)**	8.71 ± 1.18	-22.16 ± 0.76
**Girls (n=120)**	8.79 ± 1.33	-22.21 ± 0.73
	**Breastfeeding status**	
**Fully breastfed (n=7)**	11.45 ± 0.70	-22.02 ± 0.45
**Partially breastfed (n=78)**	9.73 ± 1.22**	-21.92 ± 0.68
**Fully weaned (n=170)**	8.19 ± 0.78**	-22.32 ± 0.76*
	**Wasting**	
**Not wasted (n=205)**	8.67 ± 1.22	-22.20 ± 0.78
**Wasted (n=50)**	9.08 ± 1.34*	-22.13 ± 0.61
**Severely wasted (n=18)**	9.31 ± 1.39*	-22.26 ± 0.73
	**Stunting**	
**Not stunted (n=162)**	8.89 ± 1.32	-22.14 ± 0.73
**Stunted (n=93)**	8.51 ± 1.10*	-22.28 ± 0.78
**Severely stunted (n=25)**	8.13 ± 0.85**	-22.63 ± 0.94**

$\delta^{15}N$ and $\delta^{13}C$ values (‰) are means ± SD. * and ** indicate differences from the first line of each block, $P < 0.05$ and $P < 0.01$.

Supplementary Table S4: Raw and age- and breastfeeding-adjusted odd ratios for the risk of being wasted or stunted according to hair $\delta^{15}N$ or $\delta^{13}C$ values.

		Unadjusted		Adjusted	
		OR [CI 95]	*P*	OR [CO 95]	*P*
**Wasted**	**$\delta^{15}N$**	1.28 [1.01-1.62]	0.04	1.08 [0.78-1.51]	NS
	**$\delta^{13}C$**	1.13 [0.75-1.74]	NS	0.95 [0.60-1.52]	NS
**Severely wasted**	**$\delta^{15}N$**	1.44 [1.01-2.02]	0.04	1.11 [0.70-1.78]	NS
	**$\delta^{13}C$**	0.90 [0.48-1.69]	NS	0.61 [0.29-1.24]	NS
**Stunted**	**$\delta^{15}N$**	0.77 [0.62-0.96]	0.02	0.79 [0.58-1.06]	NS
	**$\delta^{13}C$**	0.78 [0.55-1.10]	NS	0.90 [0.62-1.31]	NS
**Severely stunted**	**$\delta^{15}N$**	0.54 [0.32-0.82]	< 0.01	0.53 [0.29-0.91]	0.02
	**$\delta^{13}C$**	0.43 [0.24-0.76]	< 0.01	0.55 [0.28-1.02]	0.06

OR, odds ratio [95% CI].

Supplementary analyses

Using a mixed procedure for repeated data (PROC MIXED, SAS 9.14, Cary, NC), we assessed the change in $\delta^{15}N$ and $\delta^{13}C$ between two consecutive segments of hair, the first and second adjacent 5 mm segments closest to the scalp. The first (proximal) segment corresponds with the more recent period, from -3 to -1 weeks before sampling. The second (distal) segment corresponds with the earlier period, from -5 to -3 weeks before sampling. Because the data presented in Figure 1 and Table 4 of the manuscript suggest that the age of children at inclusion may affect the change in $\delta^{15}N$ and $\delta^{13}C$ between the two segments, age at inclusion was included as a covariate in the model.

Our results indicate a slight but significant (-0.03) decrease in both $\delta^{15}N$ and $\delta^{13}C$ from the distal to the proximal segment, corresponding to a more recent period of 2 weeks **(Supplementary Table S5)**. These results are in line with a progressive decrease in $\delta^{15}N$ and $\delta^{13}C$ observed between 6 months and 5 years, due to the weaning process.

Supplementary Table S5: $\delta^{15}N$ and $\delta^{13}C$ evolution between two consecutive hair segments

Fixed effects of the mixed model[1]:	$\delta^{15}N$	$\delta^{13}C$
Segment	P < 0.05	P < 0.05
Age at inclusion	P < 0.0001	P < 0.0001
Post-hoc comparisons:	Distal segment (-5 to -3 weeks) [2]	Proximal segment (-3 to -1 weeks) [2]
$\delta^{15}N$	8.78 ± 0.06	8.75 ± 0.06*
$\delta^{13}C$	-22.15 ± 0.05	-22.18 ± 0.04*

[1]Type 3 fixed effects of the mixed model

[2] $\delta^{15}N$ and $\delta^{13}C$ values (‰) are least-square means ± SE

* significantly different from the older segment, $P < 0.05$

We evaluated the effect of wasting and stunting on this isotopic change and did not find evidence of interaction between wasting or stunting and the changes in $\delta^{15}N$ and $\delta^{13}C$ between distal and proximal segments (**Supplementary Table S6**). This suggests that the low $\delta^{15}N$ and $\delta^{13}C$ values observed in severely stunted children are due to dietary or metabolic changes that occurred before the one-month period covered by the two consecutive hair segments.

Supplementary Table S6: Effect of stunting and wasting on $\delta^{15}N$ and $\delta^{13}C$ evolution between two consecutive hair segments

Model	Fixed effects and interaction mixed models[1]:	$\delta^{15}N$	$\delta^{13}C$
Model for wasting	Segment	*P* < 0.05	*P* = 0.10
	Age at inclusion	*P* < 0.0001	*P* < 0.0001
	Wasting	NS	NS
	Segment*Wasting	NS	NS
Model for severe wasting	Segment	*P* = 0.06	*P* = 0.05
	Age at inclusion	*P* < 0.0001	*P* < 0.0001
	Severe wasting	NS	NS
	Segment*Severe wasting	NS	NS
Model for stunting	Segment	*P* < 0.05	*P* < 0.05
	Age at inclusion	*P* < 0.0001	*P* < 0.0001
	Stunting	NS	NS
	Segment*Stunting	NS	NS
Model for severe stunting	Segment	*P* < 0.01	NS
	Age at inclusion	*P* < 0.0001	*P* < 0.0001
	Severe stunting	*P* < 0.01	*P* < 0.01
	Segment*Severe stunting	NS	NS

[1]Type 3 fixed effects and interaction of the mixed model.

Supplementary table S7: individual data

Data description:

- id: subject number
- sex: 1 = boy, 2 = girl
- age: age in months
- H: height or length in cm
- W: weight in kg
- MUAC: mid-upper arm circumference in cm
- group: breastfeeding group, weaned=weaned, part_bf=partially breastfed, ful_bf=fully breastfed.
- segment: proximal = 5 mm segment closest to the scalp, distal=next 5 mm segment.
- Δ15N and δ13C in ‰
- C:N ratio
- . indicates a missing data

id	sex	age	H	W	MUAC	group	segment	$\delta^{15}N$	$\delta^{13}C$	C:N ratio
1004	2	53	102.75	16.05	163.5	weaned	distal	7.56	-22.83	3.22
1004	2	53	102.75	16.05	163.5	weaned	proximal	7.43	-23.45	3.54
1006	1	29	81.95	9.55	122.5	weaned	distal	8.17	-22.53	3.01
1006	1	29	81.95	9.55	122.5	weaned	proximal	8.48	-22.92	3.12
1008	1	23	78.4	8.65	125.5	part_bf	distal	9.24	-22.76	3.06
1008	1	23	78.4	8.65	125.5	part_bf	proximal	9.32	-22.37	3.05
1010	1	28	71.25	7.3	124.5	part_bf	distal	8.41	-23.01	3.21
1010	1	28	71.25	7.3	124.5	part_bf	proximal	8.3	-22.87	3.17
1014	2	49	89.15	12.15	151	weaned	distal	8.56	-23.44	2.97
1014	2	49	89.15	12.15	151	weaned	proximal	8.09	-23.24	3.09
1016	2	37	92.25	15.65	175.5	part_bf	distal	7.78	-23.53	2.98
1016	2	37	92.25	15.65	175.5	part_bf	proximal	7.39	-23.51	3.27
1017	2	38	93.65	13.05	158.5	weaned	distal	8.57	-21.23	3.25
1017	2	38	93.65	13.05	158.5	weaned	proximal	8.65	-21.2	3.21
1019	1	56	104.35	15.45	155.5	weaned	distal	7.5	-22.42	3,00
1019	1	56	104.35	15.45	155.5	weaned	proximal	7.47	-22.45	2.99
1021	1	55	97.35	12.05	132.5	weaned	distal	7.17	-22.75	3.28
1021	1	55	97.35	12.05	132.5	weaned	proximal	7.16	-22.83	3.39
1023	1	46	90.55	11.65	147.5	weaned	distal	7.28	-22.98	3.14
1023	1	46	90.55	11.65	147.5	weaned	proximal	7.56	-22.93	3.07
1025	2	58	100.55	14.65	163.5	weaned	distal	7.72	-22.92	2.99
1025	2	58	100.55	14.65	163.5	weaned	proximal	8.05	-22.71	3.08
1026	1	21	87.35	9.45	135.5	part_bf	distal	10.08	-21.76	3.06
1026	1	21	87.35	9.45	135.5	part_bf	proximal	10.02	-21.65	2.97
1027	2	59	103.35	16.25	156.5	weaned	distal	8.06	-22.51	3.2
1027	2	59	103.35	16.25	156.5	weaned	proximal	7.7	-22.5	3.32
1031	2	30	83.45	12.05	149	weaned	distal	7.23	-21.97	2.96
1031	2	30	83.45	12.05	149	weaned	proximal	7.52	-22.5	3.39
1037	2	32	82.65	11.45	155.5	weaned	distal	7.82	-22.17	3.32
1037	2	32	82.65	11.45	155.5	weaned	proximal	7.53	-22.39	3.06
1040	2	39	95.4	14.05	149.5	weaned	distal	7.77	-22.63	3.11
1040	2	39	95.4	14.05	149.5	weaned	proximal	7.92	-22.69	3.39
1044	1	52	99.95	12.85	140.5	weaned	distal	7.62	-22.27	3.09

1044	1	52	99.95	12.85	140.5	weaned	proximal	7.42	-22.49	3.12
1046	2	34	85.95	10.95	139.5	weaned	distal	7.15	-22.84	3.08
1046	2	34	85.95	10.95	139.5	weaned	proximal	7.04	-23.31	3.35
1052	2	43	84.25	9.35	121.5	weaned	distal	8.24	-22.07	3.05
1052	2	43	84.25	9.35	121.5	weaned	proximal	8.03	-22.3	3.05
1054	2	48	94.35	11.85	139.5	weaned	distal	7.86	-23.14	3.2
1054	2	48	94.35	11.85	139.5	weaned	proximal	7.81	-22.85	3.27
1056	2	50	90.55	14.45	157.5	weaned	distal	7.01	-24.15	2.97
1056	2	50	90.55	14.45	157.5	weaned	proximal	6.99	-24.14	3.02
1060	1	39	88.35	13.45	156.5	weaned	distal	8.24	-22.27	3.19
1060	1	39	88.35	13.45	156.5	weaned	proximal	7.98	-22.59	3.24
1062	2	38	91.15	11.25	141.5	weaned	distal	8.48	-21.43	3.04
1062	2	38	91.15	11.25	141.5	weaned	proximal	8.43	-21.57	3.05
1064	1	12	71.05	8.25	137.5	part_bf	distal	9.95	-22.47	3.08
1064	1	12	71.05	8.25	137.5	part_bf	proximal	10.06	-22.27	3.14
1066	1	57	108.25	18.35	161.5	weaned	distal	8.17	-22.65	3.16
1066	1	57	108.25	18.35	161.5	weaned	proximal	7.9	-23.43	3.53
1068	1	9	68.45	8.55	136.5	part_bf	distal	12.17	-22.49	3.17
1068	1	9	68.45	8.55	136.5	part_bf	proximal	12.08	-22.39	3.13
1070	2	47	100.25	14.85	151.5	weaned	distal	9.04	-20.75	3.15
1070	2	47	100.25	14.85	151.5	weaned	proximal	8.61	-21.3	3.09
1074	1	57	108.85	16.55	161	weaned	distal	7.47	-22.74	3.07
1074	1	57	108.85	16.55	161	weaned	proximal	7.49	-22.98	3.17
1076	1	10	71.45	8.65	137.5	part_bf	distal	10.78	-22.13	3.04
1076	1	10	71.45	8.65	137.5	part_bf	proximal	10.5	-22.43	3.12
1078	1	51	112.1	19.55	171.5	weaned	distal	8.77	-22.1	3.01
1078	1	51	112.1	19.55	171.5	weaned	proximal	8.99	-21.94	3.06
1081	2	56	97.25	14.85	162.5	weaned	distal	8.63	-22.71	3.12
1081	2	56	97.25	14.85	162.5	weaned	proximal	8.42	-22.51	3.02
1086	2	33	90.15	13.55	155.5	weaned	distal	7.59	-23.31	3.13
1086	2	33	90.15	13.55	155.5	weaned	proximal	7.73	-23.56	3.12
1088	1	44	95.35	13.65	151.5	weaned	distal	8.18	-22.53	3.03
1088	1	44	95.35	13.65	151.5	weaned	proximal	7.87	-22.73	3.13
1092	1	52	98.55	15.55	151.5	weaned	distal	7.38	-23.27	3.1
1092	1	52	98.55	15.55	151.5	weaned	proximal	7.09	-23.34	3.08
1094	1	50	94.55	13.05	142.5	weaned	distal	7.3	-22.99	3.09
1094	1	50	94.55	13.05	142.5	weaned	proximal	6.82	-23.35	3.08
1095	1	36	90.15	10.05	133.5	weaned	distal	8.63	-22.16	3.05
1095	1	36	90.15	10.05	133.5	weaned	proximal	8.51	-22.47	2.95
1096	2	22	77.25	9.45	139.5	part_bf	distal	9.09	-21.57	3.15
1096	2	22	77.25	9.45	139.5	part_bf	proximal	8.9	-21.54	3.16
1097	2	48	100.95	13.35	149.5	weaned	distal	8.2	-21.7	3.13
1097	2	48	100.95	13.35	149.5	weaned	proximal	8.03	-21.67	3.13
1099	2	29	83.25	9.35	126.5	weaned	distal	8.38	-22.62	3.02
1099	2	29	83.25	9.35	126.5	weaned	proximal	8.61	-22.51	3.1
1103	2	58	96.05	12.15	142.5	weaned	distal	8.94	-21,00	3.04
1103	2	58	96.05	12.15	142.5	weaned	proximal	8.82	-20.86	3.22
1105	1	19	77.45	9.65	146.5	part_bf	distal	10.55	-20.84	3.18
1105	1	19	77.45	9.65	146.5	part_bf	proximal	10.74	-20.69	3.2

1109	2	7	71.05	9.55	162.5	ful_bf	distal	11.34	-21.79	3.23
1109	2	7	71.05	9.55	162.5	ful_bf	proximal	11.31	-21.85	3.28
1111	1	20	83.45	11.15	143.5	part_bf	distal	8.32	-21.55	3.43
1111	1	20	83.45	11.15	143.5	part_bf	proximal	8.45	-21.18	3.25
1113	2	15	76.15	9.05	131.5	weaned	distal	10.6	-22.86	3.59
1113	2	15	76.15	9.05	131.5	weaned	proximal	10.38	-22.99	3.74
1119	1	28	88.65	12.15	147.5	part_bf	distal	9.27	-22.49	3.11
1119	1	28	88.65	12.15	147.5	part_bf	proximal	9.13	-22.44	3.01
1121	1	38	93.25	12.35	143.5	weaned	distal	8.66	-22.5	3.15
1121	1	38	93.25	12.35	143.5	weaned	proximal	8.79	-22.58	2.97
1123	1	56	99.15	13.35	139.5	weaned	distal	7.71	-23.8	3.05
1123	1	56	99.15	13.35	139.5	weaned	proximal	7.67	-23.79	3.08
1132	1	15	74.25	7.05	114	part_bf	distal	10.33	-22.49	3.06
1132	1	15	74.25	7.05	114	part_bf	proximal	10.23	-22.53	3.35
1133	1	27	83.6	12.15	151.5	weaned	distal	8.4	-22.6	3.2
1133	1	27	83.6	12.15	151.5	weaned	proximal	7.86	-23.24	3.34
1134	1	48	94.55	12.4	143.5	weaned	distal	8.52	-22.76	3.06
1134	1	48	94.55	12.4	143.5	weaned	proximal	8.45	-22.88	3.02
1140	1	39	88.25	10.15	135.5	weaned	distal	9.09	-22.05	3.07
1140	1	39	88.25	10.15	135.5	weaned	proximal	9.19	-22.15	3.2
1142	2	54	97.85	11.95	128.5	weaned	distal	7.95	-22.31	3.15
1142	2	54	97.85	11.95	128.5	weaned	proximal	7.98	-22.19	3.14
1144	2	52	89.45	10.25	134.5	weaned	distal	7.8	-22.85	3.1
1144	2	52	89.45	10.25	134.5	weaned	proximal	7.55	-22.66	3.11
1148	2	48	95.05	12.9	141.5	weaned	distal	8.26	-22.13	3.13
1148	2	48	95.05	12.9	141.5	weaned	proximal	8.25	-22.64	3.31
1154	1	11	72.05	9.25	158.5	part_bf	distal	10.69	-21.7	3.06
1154	1	11	72.05	9.25	158.5	part_bf	proximal	10.57	-22.1	3.03
1156	2	32	87.05	13.55	164.5	weaned	distal	9.94	-22.14	3.17
1156	2	32	87.05	13.55	164.5	weaned	proximal	9.89	-22.41	3.01
1163	1	15	72.85	8.15	125.5	part_bf	distal	10.77	-21.3	3.23
1163	1	15	72.85	8.15	125.5	part_bf	proximal	10.88	-21.62	3.27
1165	2	26	76.45	8.25	129.5	weaned	distal	8.48	-21.12	3.16
1165	2	26	76.45	8.25	129.5	weaned	proximal	8.55	-21.37	3.17
1169	1	49	92.25	11.85	134.5	weaned	distal	8.65	-21.4	3.12
1169	1	49	92.25	11.85	134.5	weaned	proximal	8.55	-21.56	3.05
1173	2	43	87.75	10.7	137.5	weaned	distal	8.16	-21.62	3.08
1173	2	43	87.75	10.7	137.5	weaned	proximal	8.14	-21.71	3.32
1175	2	6	61.65	7.85	152.5	part_bf	distal	8.24	-22.97	3.84
1175	2	6	61.65	7.85	152.5	part_bf	proximal	8.58	-21.97	3.19
1181	1	54	100.15	14.95	155.5	weaned	distal	7.58	-22.92	3.04
1181	1	54	100.15	14.95	155.5	weaned	proximal	7.17	-23.07	3.01
1184	2	47	97.55	13.7	146.5	weaned	distal	8.09	-22.98	3.48
1184	2	47	97.55	13.7	146.5	weaned	proximal	8.02	-22.54	3.31
1187	2	48	89.75	12.3	146.5	weaned	distal	8.43	-21.02	3.03
1187	2	48	89.75	12.3	146.5	weaned	proximal	8.11	-21.27	3.01
2004	2	7	65.05	6.8	135	part_bf	distal	7.25	-23.22	3.05
2004	2	7	65.05	6.8	135	part_bf	proximal	6.97	-23.84	3.54
2007	1	49	100.8	14.4	154.5	weaned	distal	8.31	-22.49	3.1

2007	1	49	100.8	14.4	154.5	weaned	proximal	8.2	-22.54	3.24
2010	2	50	90.2	12.65	176.5	weaned	distal	8.46	-24.53	2.99
2010	2	50	90.2	12.65	176.5	weaned	proximal	8.29	-24.26	3.15
2012	1	53	102.45	14.2	137.5	weaned	distal	7.63	-22.51	3.08
2012	1	53	102.45	14.2	137.5	weaned	proximal	7.32	-22.38	3.14
2014	2	31	84.45	9.6	147	part_bf	distal	9.67	-22.7	3.01
2014	2	31	84.45	9.6	147	part_bf	proximal	9.8	-22.61	2.98
2019	2	23	84.15	10.2	143.5	weaned	distal	8.66	-20.98	2.98
2019	2	23	84.15	10.2	143.5	weaned	proximal	8.63	-20.77	3.14
2021	1	46	91.85	10.6	129	weaned	distal	8.14	-21.38	3,00
2021	1	46	91.85	10.6	129	weaned	proximal	8.21	-21.65	3.02
2022	1	55	97.55	13.2	143.5	weaned	distal	7.89	-22.17	3.05
2022	1	55	97.55	13.2	143.5	weaned	proximal	7.73	-22.47	3.04
2025	1	52	97.1	11.6	140	weaned	distal	7.45	-22.88	3.34
2025	1	52	97.1	11.6	140	weaned	proximal	7.69	-22.06	3.09
2027	1	46	88.95	11.7	148	weaned	distal	7.08	-22.59	3.28
2027	1	46	88.95	11.7	148	weaned	proximal	7.29	-22.72	2.98
2029	2	52	88.15	11.3	138.5	weaned	distal	8.1	-23.07	3.03
2029	2	52	88.15	11.3	138.5	weaned	proximal	8.01	-23.48	3.43
2031	1	40	87.85	11.25	141.5	weaned	distal	8.62	-21.77	3.29
2031	1	40	87.85	11.25	141.5	weaned	proximal	8.48	-22.17	3.13
2033	1	57	100.15	14.85	152.5	weaned	distal	8.39	-20.72	3.25
2033	1	57	100.15	14.85	152.5	weaned	proximal	8.25	-20.77	3.25
2034	2	30	82.85	11.7	157	weaned	distal	7.72	-21.16	3.19
2034	2	30	82.85	11.7	157	weaned	proximal	7.93	-21.59	3.02
2036	1	35	85.6	11.2	141.5	part_bf	distal	8.36	-22.69	3.12
2036	1	35	85.6	11.2	141.5	part_bf	proximal	8.19	-22.52	3.11
2040	1	57	105.3	14.4	147.5	weaned	distal	8.62	-22.5	3.09
2040	1	57	105.3	14.4	147.5	weaned	proximal	8.05	-22.47	3.14
2042	1	37	96.5	14.2	157	weaned	distal	8.5	-23.75	3.16
2042	1	37	96.5	14.2	157	weaned	proximal	8.7	-23.99	3.05
2044	1	18	77.4	7.9	123	part_bf	distal	10.15	-21.27	3.3
2044	1	18	77.4	7.9	123	part_bf	proximal	10.07	-21.72	3.9
2047	1	50	98.4	14.7	162.5	weaned	distal	8.25	-20.59	3.15
2047	1	50	98.4	14.7	162.5	weaned	proximal	8.21	-20.7	3.13
2049	1	58	98.3	12.4	137	weaned	distal	8.49	-21.37	3.13
2049	1	58	98.3	12.4	137	weaned	proximal	8.33	-21.29	3.08
2051	1	29	84.9	10.2	143	weaned	distal	8.21	-20.9	3.27
2051	1	29	84.9	10.2	143	weaned	proximal	8.06	-20.78	3.15
2053	2	27	76.6	7.9	123	part_bf	distal	9.37	-21.97	3.11
2053	2	27	76.6	7.9	123	part_bf	proximal	9.52	-22.23	3.09
2055	1	29	89.1	11.7	146	part_bf	distal	8.89	-20.54	3.14
2055	1	29	89.1	11.7	146	part_bf	proximal	8.81	-20.79	3.22
2057	1	46	82.85	10.7	137	weaned	distal	6.91	-23.85	3.15
2057	1	46	82.85	10.7	137	weaned	proximal	6.6	-24.15	3.1
2059	1	25	80.55	10.45	147	part_bf	distal	10.31	-21.91	3.13
2059	1	25	80.55	10.45	147	part_bf	proximal	10.5	-21.72	3.1
2061	2	28	81.4	8.9	122.5	weaned	distal	8.84	-21.52	3.02
2061	2	28	81.4	8.9	122.5	weaned	proximal	8.84	-21.11	3.03

2063	2	24	79.3	7.85	123	part_bf	distal	10.82	-21.56	3.09
2063	2	24	79.3	7.85	123	part_bf	proximal	10.7	-21.74	3.07
2064	2	10	70.9	6.2	114	part_bf	distal	9.99	-21.12	3.02
2064	2	10	70.9	6.2	114	part_bf	proximal	10.12	-20.85	3.13
2065	2	28	84.65	10.35	141	part_bf	distal	10.48	-22.81	3.01
2065	2	28	84.65	10.35	141	part_bf	proximal	10.44	-22.81	3.08
2067	1	24	78.3	9.8	148	weaned	distal	9.67	-21.86	3.01
2067	1	24	78.3	9.8	148	weaned	proximal	9.82	-21.78	3.1
2072	1	55	98.3	13.3	136.5	weaned	distal	8.97	-21.88	3.14
2072	1	55	98.3	13.3	136.5	weaned	proximal	8.84	-21.78	3.09
2074	1	22	81.2	9.8	130.5	part_bf	distal	10.11	-21.61	3.07
2074	1	22	81.2	9.8	130.5	part_bf	proximal	10.03	-21.55	3.06
2076	1	31	96.6	12	134	weaned	distal	8.29	.	3.02
2076	1	31	96.6	12	134	weaned	proximal	7.9	.	3.02
2080	2	9	68.8	8	143	ful_bf	distal	12.46	-22.09	3.22
2080	2	9	68.8	8	143	ful_bf	proximal	12.97	-21.86	3.13
2083	1	28	83.35	11.7	154	part_bf	distal	9.28	-22.09	3.25
2083	1	28	83.35	11.7	154	part_bf	proximal	9.32	-22.07	3.14
2085	1	40	80.9	10.9	145	weaned	distal	8.01	.	3.01
2085	1	40	80.9	10.9	145	weaned	proximal	7.76	.	3,00
2087	2	23	81.2	10.8	143	weaned	distal	8.52	-21.78	3.14
2087	2	23	81.2	10.8	143	weaned	proximal	8.53	-22.21	2.98
2089	2	23	79.3	8.7	120	weaned	distal	8.86	-21.53	2.99
2089	2	23	79.3	8.7	120	weaned	proximal	8.72	-21.52	3.1
2091	1	25	77.85	8.6	139	weaned	distal	10.43	-21.56	3.03
2091	1	25	77.85	8.6	139	weaned	proximal	10.37	-21.56	3.04
2093	1	26	82.2	9.1	136	weaned	distal	7.53	-21.18	3.07
2093	1	26	82.2	9.1	136	weaned	proximal	7.66	-21.02	2.98
2099	1	15	82.2	14.2	186	part_bf	distal	9,00	-20.01	3.07
2099	1	15	82.2	14.2	186	part_bf	proximal	8.91	-20.53	2.99
2101	1	14	72.1	8.1	139	part_bf	distal	11.32	-21.42	3.07
2101	1	14	72.1	8.1	139	part_bf	proximal	11.27	-21.38	3.09
2102	1	7	69.5	8.3	142	ful_bf	distal	11.2	-21.84	3.11
2102	1	7	69.5	8.3	142	ful_bf	proximal	11.11	-21.94	3.1
2104	2	54	99.6	14.2	145	weaned	distal	8.22	-22.23	3.11
2104	2	54	99.6	14.2	145	weaned	proximal	8.17	-22.33	3.16
2105	1	12	72.6	6.9	124	part_bf	distal	9.94	-21.44	3.04
2105	1	12	72.6	6.9	124	part_bf	proximal	9.25	-22.18	3.85
2107	2	12	74.1	7.6	127	part_bf	distal	11.76	-22.1	3.08
2107	2	12	74.1	7.6	127	part_bf	proximal	11.82	-22.17	2.98
2109	1	48	100.1	14.3	155	weaned	distal	8.53	-22.37	3.1
2109	1	48	100.1	14.3	155	weaned	proximal	8.66	-22.24	3.1
2113	2	19	77.8	8.3	124	part_bf	distal	8.53	-22.13	3.12
2113	2	19	77.8	8.3	124	part_bf	proximal	8.26	-22.38	3.1
2115	1	39	87.5	9.5	129	weaned	distal	8.03	-22.27	2.96
2115	1	39	87.5	9.5	129	weaned	proximal	.	.	.
2127	1	7	67.8	7.3	131	part_bf	distal	10.38	-21.75	3.13
2127	1	7	67.8	7.3	131	part_bf	proximal	10.16	-21.95	2.98
2129	2	28	85.85	10.7	144	weaned	distal	9.14	-22.78	3.01

2129	2	28	85.85	10.7	144	weaned	proximal	9.12	-22.63	3.05
2131	1	50	91.5	12.3	152	weaned	distal	7.51	-22.65	3.08
2131	1	50	91.5	12.3	152	weaned	proximal	7.67	-22.52	3.06
2133	1	33	87.9	10.9	146	weaned	distal	8.87	-22.05	3.08
2133	1	33	87.9	10.9	146	weaned	proximal	8.82	-22.02	3.04
2135	2	33	86.8	11.3	152	weaned	distal	7.88	-22.93	3.1
2135	2	33	86.8	11.3	152	weaned	proximal	8.23	-22.7	3.13
2137	1	26	87.1	11.2	147	weaned	distal	9.07	-22.12	3.14
2137	1	26	87.1	11.2	147	weaned	proximal	8.64	-22.56	3.06
2139	1	28	87.6	12.2	155	weaned	distal	8.51	-21.36	3.13
2139	1	28	87.6	12.2	155	weaned	proximal	8.11	-21.56	3,00
2141	2	54	99.35	15	162	weaned	distal	8.73	-21.67	3,00
2141	2	54	99.35	15	162	weaned	proximal	8.66	-21.82	3.08
2143	2	28	93.4	13.2	161	weaned	distal	7.77	-21.34	3.11
2143	2	28	93.4	13.2	161	weaned	proximal	8.02	-21.4	3.22
2145	1	8	72.1	9.3	156.5	part_bf	distal	10.55	-21.31	3.06
2145	1	8	72.1	9.3	156.5	part_bf	proximal	10.11	-21.13	3.12
2147	1	20	78	8.5	133	part_bf	distal	11.74	-22.07	3.19
2147	1	20	78	8.5	133	part_bf	proximal	11.61	-21.97	3.11
2148	2	9	65.6	4.3	82	ful_bf	distal	10.43	-22.89	3.03
2148	2	9	65.6	4.3	82	ful_bf	proximal	11,00	-22.86	3,00
2150	2	28	77.3	9	144	weaned	distal	8.05	-23.44	3.01
2150	2	28	77.3	9	144	weaned	proximal	8.05	-23.49	3.22
2156	1	49	99.5	13.5	147	weaned	distal	8.34	-21.49	3.09
2156	1	49	99.5	13.5	147	weaned	proximal	8.28	-21.56	3.15
2157	1	48	99.1	11.8	130.5	weaned	distal	8.87	-21.12	3.14
2157	1	48	99.1	11.8	130.5	weaned	proximal	8.56	-21.51	3.25
2158	2	13	73	8.1	141	ful_bf	distal	11.61	-21.52	3,00
2158	2	13	73	8.1	141	ful_bf	proximal	11.28	-21.42	3.14
2159	2	7	63.4	5	111.5	part_bf	distal	10.4	-21.52	3.07
2159	2	7	63.4	5	111.5	part_bf	proximal	10.4	-21.18	3.03
2160	2	21	74.2	7.7	131	weaned	distal	7.35	-22.02	3.17
2160	2	21	74.2	7.7	131	weaned	proximal	7.19	-21.5	3.14
2162	1	27	84.2	9.9	142	weaned	distal	9.6	-20.55	2.98
2162	1	27	84.2	9.9	142	weaned	proximal	9.65	-20.53	3.1
2164	1	27	82	10	140.5	weaned	distal	8.81	-20.54	3.11
2164	1	27	82	10	140.5	weaned	proximal	8.93	-20.77	3.08
2166	1	43	96.7	12.2	141	weaned	distal	7.36	-21.91	3.12
2166	1	43	96.7	12.2	141	weaned	proximal	7.68	-21.93	3.09
2167	2	42	96.2	10.8	124	weaned	distal	8.05	-23.05	2.98
2167	2	42	96.2	10.8	124	weaned	proximal	7.83	-22.91	3.04
2169	1	11	71.2	7	123	part_bf	distal	10.79	-21.67	3.22
2169	1	11	71.2	7	123	part_bf	proximal	10.46	-21.66	3.08
2171	1	14	78.9	8.9	132	part_bf	distal	10.3	-22.02	3.07
2171	1	14	78.9	8.9	132	part_bf	proximal	10.15	-22.07	3.22
2175	2	40	90.55	12.5	146	weaned	distal	7.94	-23.64	3.07
2175	2	40	90.55	12.5	146	weaned	proximal	7.98	-23.14	3.04
2177	1	20	79	9.5	125	part_bf	distal	10.41	-21.4	3.13
2177	1	20	79	9.5	125	part_bf	proximal	10.32	-21.45	3.07

2179	1	51	98.4	13.4	143.5	weaned	distal	8.12	-21.62	3.01
2179	1	51	98.4	13.4	143.5	weaned	proximal	8.11	-21.64	2.98
2182	1	59	101.2	14.2	146	weaned	distal	8.47	-21.82	3.25
2182	1	59	101.2	14.2	146	weaned	proximal	8.61	-21.8	3.14
3008	1	51	92.85	10.5	128.5	weaned	distal	8.39	-23.26	3,00
3008	1	51	92.85	10.5	128.5	weaned	proximal	8.52	-23.58	2.95
3009	2	52	93.05	12.6	150.5	weaned	distal	8.42	-22.57	3.35
3009	2	52	93.05	12.6	150.5	weaned	proximal	8.55	-22.35	3.41
3013	2	31	82.2	11.45	147.5	weaned	distal	6.36	-22.62	2.95
3013	2	31	82.2	11.45	147.5	weaned	proximal	6.39	-22.7	2.96
3015	2	13	74.85	9.1	135.5	part_bf	distal	10.14	-20.96	3.14
3015	2	13	74.85	9.1	135.5	part_bf	proximal	10.27	-20.99	3.15
3017	2	37	88.2	12.1	152.5	weaned	distal	7.83	-22.3	3.14
3017	2	37	88.2	12.1	152.5	weaned	proximal	7.82	-21.77	2.95
3019	2	52	95.45	13.05	155	weaned	distal	11.55	-22.64	3.26
3019	2	52	95.45	13.05	155	weaned	proximal	11.74	-22.65	3.01
3021	1	40	98.15	13.4	154	weaned	distal	8.87	-22.1	3.11
3021	1	40	98.15	13.4	154	weaned	proximal	8.87	-22.12	3.07
3023	1	18	84.15	11.75	145.5	part_bf	distal	10.2	-22.06	3.03
3023	1	18	84.15	11.75	145.5	part_bf	proximal	10.17	-22.03	3.11
3024	1	7	66.55	6.4	120	part_bf	distal	10.48	-21.79	3.11
3024	1	7	66.55	6.4	120	part_bf	proximal	10.59	-21.82	3.19
3026	2	17	77.2	9.6	154	part_bf	distal	10.03	-22.16	3.15
3026	2	17	77.2	9.6	154	part_bf	proximal	9.88	-21.89	3.12
3028	2	30	89.25	13.3	157	weaned	distal	.	.	.
3028	2	30	89.25	13.3	157	weaned	proximal	8.32	-22.35	3.16
3030	2	7	62.35	6.1	120	part_bf	distal	11.27	-22.27	3.25
3030	2	7	62.35	6.1	120	part_bf	proximal	11.02	-22.22	3.26
3032	2	32	86.75	11.45	149	weaned	distal	8.79	-21.76	3.03
3032	2	32	86.75	11.45	149	weaned	proximal	8.78	-21.4	3.03
3035	1	36	86.6	10.9	146.5	part_bf	distal	8.45	-22.68	3.02
3035	1	36	86.6	10.9	146.5	part_bf	proximal	8.11	-22.33	3.26
3037	2	7	65.15	6.05	113	part_bf	distal	12.22	-21.89	3.25
3037	2	7	65.15	6.05	113	part_bf	proximal	12.52	-21.99	3.18
3041	1	36	88.35	11.65	147.5	weaned	distal	7.9	-22.64	3.05
3041	1	36	88.35	11.65	147.5	weaned	proximal	8.03	-22.65	3.18
3043	2	33	89.2	11.2	144.5	weaned	distal	8.16	-22.94	3.06
3043	2	33	89.2	11.2	144.5	weaned	proximal	8.38	-22.58	3.24
3045	2	25	79.6	10.6	147	part_bf	distal	7.93	-21.8	3.19
3045	2	25	79.6	10.6	147	part_bf	proximal	7.96	-21.68	3.28
3047	1	11	74.35	7.8	124	part_bf	distal	10,00	-21.51	3.24
3047	1	11	74.35	7.8	124	part_bf	proximal	10.09	-21.55	3.16
3049	2	22	65.25	8.4	133	weaned	distal	8.18	-21.54	2.97
3049	2	22	65.25	8.4	133	weaned	proximal	8.05	-21.45	3.13
3051	2	40	89.1	10.8	138.5	weaned	distal	7.67	-21.04	3.22
3051	2	40	89.1	10.8	138.5	weaned	proximal	7.95	-21.14	2.97
3053	1	45	90.1	12.8	145	weaned	distal	7.68	-22.85	3.19
3053	1	45	90.1	12.8	145	weaned	proximal	7.48	-23.12	3.14
3059	1	30	77.6	8.6	127.5	part_bf	distal	8.8	-21.49	3.36

3059	1	30	77.6	8.6	127.5	part_bf	proximal	8.65	-21.37	3.27
3061	1	19	74.85	8.7	139.5	part_bf	distal	9.54	-22.92	3.09
3061	1	19	74.85	8.7	139.5	part_bf	proximal	9.23	-22.88	3.16
3063	1	54	103.05	13.2	137.5	weaned	distal	7.42	-22.64	3.02
3063	1	54	103.05	13.2	137.5	weaned	proximal	7.43	-22.58	3.01
3066	2	52	98.45	12.8	140.5	weaned	distal	7.84	-23.12	3.01
3066	2	52	98.45	12.8	140.5	weaned	proximal	7.64	-22.76	3.1
3068	1	42	96.2	14.55	160.5	weaned	distal	8.28	-22.36	3.1
3068	1	42	96.2	14.55	160.5	weaned	proximal	8.31	-22.19	3.08
3070	1	24	81.3	9.85	133.5	part_bf	distal	8.32	-21.06	3.08
3070	1	24	81.3	9.85	133.5	part_bf	proximal	8.16	-20.92	3.16
3074	1	39	91.15	14.1	161.5	weaned	distal	7.36	-20.98	2.99
3074	1	39	91.15	14.1	161.5	weaned	proximal	7.68	-21.36	3.13
3076	1	21	81.3	6.35	129.5	part_bf	distal	10.01	-21.75	2.96
3076	1	21	81.3	6.35	129.5	part_bf	proximal	9.73	-21.51	3.01
3078	1	27	85.2	11.25	142.5	weaned	distal	6.87	-23.11	3.02
3078	1	27	85.2	11.25	142.5	weaned	proximal	7.02	-23.01	3.28
3081	2	55	96.3	10.7	144.5	weaned	distal	8.78	-23.07	2.98
3081	2	55	96.3	10.7	144.5	weaned	proximal	8.83	-23,00	2.99
3084	1	27	82.1	10.45	139	part_bf	distal	9.76	-21.65	3.01
3084	1	27	82.1	10.45	139	part_bf	proximal	9.66	-21.77	3.01
3086	2	34	84.3	11.1	148.5	weaned	distal	8.66	-22.26	3.16
3086	2	34	84.3	11.1	148.5	weaned	proximal	8.69	-22.13	3.1
3088	2	31	85.1	10.3	138.5	part_bf	distal	10.69	-22.24	3.24
3088	2	31	85.1	10.3	138.5	part_bf	proximal	10.55	-22.44	3.11
3093	2	38	88.35	12.7	152.5	weaned	distal	8.73	-21.03	3.23
3093	2	38	88.35	12.7	152.5	weaned	proximal	9.08	-21.18	3.08
3095	1	14	84.35	12.85	164.5	part_bf	distal	9.27	-21.48	3.2
3095	1	14	84.35	12.85	164.5	part_bf	proximal	9.42	-21.21	3,00
3101	2	28	81.95	10.35	139.5	part_bf	distal	9.88	-21.6	3.04
3101	2	28	81.95	10.35	139.5	part_bf	proximal	9.76	-21.92	3.02
3104	2	39	89.1	11	135.5	weaned	distal	6.94	-22.47	.
3104	2	39	89.1	11	135.5	weaned	proximal	6.94	-22.47	3.13
3106	2	17	77.45	9.55	143.5	part_bf	distal	9.96	-22.52	3.04
3106	2	17	77.45	9.55	143.5	part_bf	proximal	9.44	-22.45	3.2
3108	1	14	66.65	7.15	123.5	part_bf	distal	9.63	-22.02	3,00
3108	1	14	66.65	7.15	123.5	part_bf	proximal	9.49	-22.43	3.05
3112	2	10	69.15	6.95	131	ful_bf	distal	11.23	-22.33	3.36
3112	2	10	69.15	6.95	131	ful_bf	proximal	11.52	-22.25	3.05
3114	1	58	101.35	16.45	127.5	weaned	distal	8.76	-23.22	3.06
3114	1	58	101.35	16.45	127.5	weaned	proximal	8.34	-23.26	3.08
3116	2	9	71.85	8.3	142	part_bf	distal	9.89	-20.85	3.06
3116	2	9	71.85	8.3	142	part_bf	proximal	9.9	-21.01	3.15
3118	2	25	79.25	9.75	140.5	weaned	distal	9.59	-22.95	3.03
3118	2	25	79.25	9.75	140.5	weaned	proximal	9.34	-22.87	3.13
3121	2	22	77.45	9.45	136.5	weaned	distal	9.59	-21.93	3.08
3121	2	22	77.45	9.45	136.5	weaned	proximal	8.7	-22.04	3.08
3125	2	21	79.65	10.2	142.5	part_bf	distal	10.94	-21.47	3.21
3125	2	21	79.65	10.2	142.5	part_bf	proximal	10.83	-21.73	3.03

3127	1	22	82.85	10.85	142.5	part_bf	distal	10.6	-21.83	3.12
3127	1	22	82.85	10.85	142.5	part_bf	proximal	10.71	-21.9	3.02
3132	1	20	77.65	9.45	131	part_bf	distal	9.04	-21.5	3.12
3132	1	20	77.65	9.45	131	part_bf	proximal	9.05	-21.37	3.02
3133	2	6	62.45	5.4	113	part_bf	distal	10.86	-22.52	2.98
3133	2	6	62.45	5.4	113	part_bf	proximal	10.94	-22.84	3.15
3143	1	8	70.1	6.85	123	part_bf	distal	11.63	-22.87	3.11
3143	1	8	70.1	6.85	123	part_bf	proximal	11.8	-22.74	3.09
3146	1	38	85.25	11.45	140.5	weaned	distal	7.7	-23.19	3.12
3146	1	38	85.25	11.45	140.5	weaned	proximal	8.19	-23.68	3.1
3148	2	44	98.3	13.95	143	weaned	distal	7.66	-22.16	3.04
3148	2	44	98.3	13.95	143	weaned	proximal	7.55	-22,00	3.03
4004	1	51	98.65	15.05	161.5	weaned	distal	8.05	-22.46	3.23
4004	1	51	98.65	15.05	161.5	weaned	proximal	7.79	-22.54	3.22
4006	1	41	92.15	15.5	157	weaned	distal	8.24	-22.25	3.24
4006	1	41	92.15	15.5	157	weaned	proximal	8.14	-22.08	3,00
4008	1	40	96.65	14.25	167	weaned	distal	8.94	-22.53	3.06
4008	1	40	96.65	14.25	167	weaned	proximal	9.23	-22.3	3.01
4010	1	34	103.35	11.6	136	weaned	distal	8.35	-21.77	3.09
4010	1	34	103.35	11.6	136	weaned	proximal	8.08	-22.06	2.92
4011	2	52	103.35	14.5	156.5	weaned	distal	8.25	-21.59	3.1
4011	2	52	103.35	14.5	156.5	weaned	proximal	8.41	-21.59	3.05
4014	2	11	97.7	7.15	100	part_bf	distal	7.68	-22.14	3.12
4014	2	11	97.7	7.15	100	part_bf	proximal	7.52	-22.1	3,00
4015	2	45	92.65	11.6	140	weaned	distal	8.61	-21.54	3.14
4015	2	45	92.65	11.6	140	weaned	proximal	8.82	-21.79	3.2
4018	2	21	76.45	9.4	138.5	part_bf	distal	9.67	-22.13	3.1
4018	2	21	76.45	9.4	138.5	part_bf	proximal	9.96	-22.28	3.16
4020	2	17	71.75	7.35	126.5	part_bf	distal	9.72	-22.31	4.07
4020	2	17	71.75	7.35	126.5	part_bf	proximal	9.83	-22.65	4.17
4022	2	52	101.9	14.5	152.5	weaned	distal	7.8	-21.83	3.12
4022	2	52	101.9	14.5	152.5	weaned	proximal	7.97	-21.86	3.1
4026	2	49	102.95	15.6	157.5	weaned	distal	8.73	-21.26	3.01
4026	2	49	102.95	15.6	157.5	weaned	proximal	8.49	-21.2	3.17
4028	1	50	96.05	13.6	146	weaned	distal	7.44	-22.8	3.24
4028	1	50	96.05	13.6	146	weaned	proximal	7.67	-22.59	2.99
4033	1	55	95.65	11.6	135	weaned	distal	8.05	-22.47	2.98
4033	1	55	95.65	11.6	135	weaned	proximal	7.92	-22.32	3.13
4036	1	52	104.65	15.9	156.5	weaned	distal	7.42	-23.74	3.22
4036	1	52	104.65	15.9	156.5	weaned	proximal	6.91	-23.63	3.17
4038	1	26	80.25	9.8	131.5	part_bf	distal	9.15	-23.39	3.21
4038	1	26	80.25	9.8	131.5	part_bf	proximal	9.31	-23.08	3.21
4040	2	33	86.35	12.1	155	weaned	proximal	7.79	-23.14	3.06
4041	1	28	93.35	13	146.5	weaned	distal	8.32	-22.99	3,00
4041	1	28	93.35	13	146.5	weaned	proximal	8.27	-22.96	3.02
4043	1	17	80.1	10.3	139	part_bf	distal	8.21	-22.69	3.04
4043	1	17	80.1	10.3	139	part_bf	proximal	8.19	-22.75	3.04
4046	2	31	88.15	11.5	140.5	weaned	distal	7.65	-22.26	3.2
4046	2	31	88.15	11.5	140.5	weaned	proximal	7.68	-22.88	3.21

4048	2	59	109.3	16.05	154	weaned	distal	6.78	-22.63	3.12
4048	2	59	109.3	16.05	154	weaned	proximal	6.56	-22.89	2.95
4050	1	45	92.3	13.2	150.5	weaned	distal	8.33	-22.92	3.17
4050	1	45	92.3	13.2	150.5	weaned	proximal	8.27	-22.88	3.15
4052	2	32	83.55	11.2	151.5	weaned	distal	7.61	-22.29	3.16
4052	2	32	83.55	11.2	151.5	weaned	proximal	7.8	-21.96	3.04
4054	1	33	88.7	10.8	132.5	weaned	distal	7.02	-22.07	3.06
4054	1	33	88.7	10.8	132.5	weaned	proximal	7.23	-22.08	3.21
4056	2	46	98.2	13.7	155.5	weaned	distal	8.5	-22.57	3.1
4056	2	46	98.2	13.7	155.5	weaned	proximal	8.12	-22.63	2.99
4058	1	42	92.25	12.3	150.5	weaned	distal	7.94	-21.41	3.16
4058	1	42	92.25	12.3	150.5	weaned	proximal	7.66	-21.95	3.18
4062	2	33	86.35	12	145.5	weaned	distal	8.3	-21.44	3.43
4062	2	33	86.35	12	145.5	weaned	proximal	8.41	-21.54	3.22
4064	1	22	80.3	9.1	136	weaned	distal	8.74	-21.27	3.1
4064	1	22	80.3	9.1	136	weaned	proximal	8.6	-21.66	3.01
4067	2	15	75.1	9.8	155	part_bf	distal	10.67	-20.94	3.33
4067	2	15	75.1	9.8	155	part_bf	proximal	10.37	-21.54	3.3
4069	1	33	88.6	11.3	149	weaned	distal	7.99	-22.36	2.98
4069	1	33	88.6	11.3	149	weaned	proximal	7.68	-22.47	3.3
4071	2	55	96.7	13.1	148.5	weaned	distal	8.4	-22.49	2.93
4071	2	55	96.7	13.1	148.5	weaned	proximal	8.6	-22.88	3.11
4073	2	55	95.25	12.5	154.5	part_bf	distal	8.21	-21.73	3.09
4073	2	55	95.25	12.5	154.5	part_bf	proximal	7.89	-21.75	3.07
4075	1	47	99.4	15.4	155.5	weaned	distal	7.89	-22.67	3.1
4075	1	47	99.4	15.4	155.5	weaned	proximal	7.95	-22.56	3.07
4077	1	28	84.25	10.7	143.5	weaned	distal	8.17	-21.31	2.96
4077	1	28	84.25	10.7	143.5	weaned	proximal	7.97	-21.32	2.96
4082	2	42	88.3	11.3	152.5	weaned	distal	7.88	-22.17	3.07
4082	2	42	88.3	11.3	152.5	weaned	proximal	7.76	-22.17	3.01
4085	2	25	83.5	11.5	152	weaned	distal	8.73	-21.24	3.11
4085	2	25	83.5	11.5	152	weaned	proximal	9.03	-20.93	3.16
4087	1	19	80.2	9.5	134.5	part_bf	distal	9.59	-21.78	4.3
4087	1	19	80.2	9.5	134.5	part_bf	proximal	10.29	-21.34	3.83
4089	1	39	93.3	12.3	152	weaned	distal	7.38	-22.62	3.01
4089	1	39	93.3	12.3	152	weaned	proximal	7.31	-22.51	3.17
4091	2	36	87.05	11.9	155.5	weaned	distal	8.28	-22.99	3.02
4091	2	36	87.05	11.9	155.5	weaned	proximal	8.5	-22.85	3.05
4093	2	49	98.75	14.1	150	weaned	distal	10.13	-23.3	3.34
4093	2	49	98.75	14.1	150	weaned	proximal	10.31	-22.28	3.9
4095	2	42	93.05	13	155.5	weaned	distal	8.44	-22.08	3.17
4095	2	42	93.05	13	155.5	weaned	proximal	8.51	-22.36	3.09
4096	1	50	95.8	12.4	145	weaned	distal	7.98	-22.65	3.14
4096	1	50	95.8	12.4	145	weaned	proximal	8.06	-22.32	3.13
4098	2	46	94.1	13	143.5	weaned	distal	8.4	-21.98	3.02
4098	2	46	94.1	13	143.5	weaned	proximal	8.41	-22.93	3.07
4100	1	27	80.65	9.7	138.5	weaned	distal	8.47	-21.58	3.03
4100	1	27	80.65	9.7	138.5	weaned	proximal	8.35	-21.36	3.28
4101	2	48	95.55	12	137.5	weaned	distal	7.78	-22.13	3.13

4101	2	48	95.55	12	137.5	weaned	proximal	7.87	-22.19	3.03
4105	2	31	81.65	11.5	153	weaned	distal	7.28	-23.08	3.14
4105	2	31	81.65	11.5	153	weaned	proximal	7.18	-22.91	3.13
4106	1	8	70.25	7.3	128.5	weaned	distal	10.97	-21.3	3.55
4106	1	8	70.25	7.3	128.5	weaned	proximal	11.13	-21.74	3.14
4108	2	47	90.65	11.45	140	weaned	distal	8.52	-22.08	2.98
4108	2	47	90.65	11.45	140	weaned	proximal	10.23	-22.02	3.69
4110	2	50	104.05	18	178.5	weaned	proximal	7.59	-22.63	3.26
4112	2	14	77.65	8.2	135	part_bf	distal	7.52	-21.92	3.17
4112	2	14	77.65	8.2	135	part_bf	proximal	7.66	-21.79	3.46
4114	1	22	80.75	10.1	142.5	weaned	distal	7.64	-23.41	2.98
4114	1	22	80.75	10.1	142.5	weaned	proximal	7.52	-23.15	3.37
4115	1	36	75	7.8	112	part_bf	distal	8.1	-22.73	3.08
4115	1	36	75	7.8	112	part_bf	proximal	7.46	-23.2	3.43
4116	1	50	102.1	15	161	weaned	distal	7.66	-23.7	3.16
4116	1	50	102.1	15	161	weaned	proximal	7.79	-23.12	3.02
4118	2	51	99.3	14	150	weaned	distal	8.87	-22.12	3.02
4118	2	51	99.3	14	150	weaned	proximal	9.16	-22.02	3.07
4120	1	18	82.25	9.6	143.5	part_bf	distal	9.27	-20.67	3.12
4120	1	18	82.25	9.6	143.5	part_bf	proximal	9.03	-20.83	2.98
4123	1	8	67.5	7	132	part_bf	distal	7.47	-20.91	2.96
4123	1	8	67.5	7	132	part_bf	proximal	7.58	-21.05	2.95
4124	1	7	66.45	7.8	146	ful_bf	distal	10.89	-21.59	3.36
4124	1	7	66.45	7.8	146	ful_bf	proximal	10.96	-21.95	3.32
4126	2	9	69.65	7.8	149	part_bf	distal	12.48	-20.94	3.25
4126	2	9	69.65	7.8	149	part_bf	proximal	12.31	-21.5	3.19
4130	2	6	69.65	8.4	156.5	part_bf	distal	11.33	-20.69	3.09
4130	2	6	69.65	8.4	156.5	part_bf	proximal	11.13	-20.94	3.26
4132	2	49	95.85	13.9	155.5	weaned	distal	8.15	-22.32	3.06
4132	2	49	95.85	13.9	155.5	weaned	proximal	7.93	-22.31	3.19
4134	1	47	89.75	12.7	151	weaned	distal	8.13	-22.23	3.12
4134	1	47	89.75	12.7	151	weaned	proximal	8.33	-22.18	3.15
4140	1	52	94.3	13.8	153.5	weaned	distal	7.63	-22.26	3.03
4140	1	52	94.3	13.8	153.5	weaned	proximal	7.82	-22.23	3.13
